# The effects of contextual bias on face recognition decisions

**DOI:** 10.1111/1556-4029.70177

**Published:** 2025-09-09

**Authors:** Lee J. Curley, Emily Breese, James Munro, Catriona Havard, Faye Skelton, Graham Pike

**Affiliations:** ^1^ Department of Psychology Glasgow Caledonian University Glasgow Scotland; ^2^ Faculty of Arts and Social Sciences, School of Psychology and Counselling The Open University Milton Keynes UK; ^3^ The School of Applied Science Edinburgh Napier University Edinburgh Scotland

**Keywords:** cognitive bias, confirmation bias, contextual bias, forensic decision‐making, prosopagnosia, super‐recognizers

## Abstract

Contemporary research has demonstrated the effects of bias on, even expert, forensic decision making. The paper aimed to test if forensically relevant face recognition decisions could be influenced by biasing information. A 3 (Bias (within‐subjects): positive bias vs. negative bias vs. control) × 2 (evidence strength (between‐subjects): weak video evidence (*N* = 97) vs. strong video evidence (*N* = 98)) × 2 (target presence (within‐subjects): absent vs. present) mixed‐design was utilized. Confidence, accuracy, and decision time were measured. In total, 195 participants were recruited. The Cambridge face memory test+ was used to measure face recognition ability. Participants saw 36 videos emulating Closed Circuit Television (CCTV) footage of a person walking down the corridor. Participants were randomly allocated to either the strong or weak evidence condition. Participants were shown a statement for each video that contained either a positive bias (target face matched the face in the video), a negative bias (target face did not match the face in the video), or control (no statement provided). Participants were then presented with a target face and asked if it matched the face seen in the previous video. There was a significant interaction between the bias and the target presence factors, with accuracy and confidence increasing and decision times decreasing when a positive bias statement was used when the target was present. Face recognition abilities did not act as a covariate. Bias may influence facial recognition decisions, and superior face recognition abilities do not undermine the influence of bias. Recommendations/implications, such as linear sequential unmasking, were discussed.

**Highlights**

- Contextual bias was shown to influence facial recognition decision‐making.
- Decision time, accuracy, and confidence impacted by congruency with the target present condition.
- Facial recognition ability (i.e., level of expertise) did not attenuate the role of bias in decision‐making.
- Implications and recommendations for police and facial recognition decision‐making are discussed.

## 1 INTRODUCTION

### 1.1 Contextual bias effects on forensic evidence

Research investigating the effects of contextual bias on forensic judgments is still in its infancy [1, 2, 3, 4, 5, 6, 7, 8]. Despite this, the field has increased dramatically over the last decade and has provided evidence to show that forensic scientists can be influenced by non‐relevant contextual information when making decisions. For instance, when contextual information was presented to forensic scientists that biased them away from deciding on a fingerprint match (i.e., knowledge of erroneous match in a prior evaluation), they were likely to change their previous decision of a match to either a non‐match or a “cannot decide” outcome [9]. Similarly, Dror and Hampikian [3] found that when forensic scientists were analyzing ambiguous DNA samples that they were also susceptible to cognitive bias. Dror and colleagues' work does not stand in isolation; Cooper and Meterko [1] conducted a systematic review and highlighted that “29 studies in 14 disciplines [of forensic science] demonstrate influence of confirmation bias” (p. 1). Previous research has shown that decision making is never fully objective, and individuals are likely to be influenced by biasing information (such as, most relevant to the current paper, knowledge of a previous decision).

Two of the key elements that have been shown to affect whether bias influences decisions are ambiguity and expertise. First, the decisions of individuals are much more likely to be influenced by biasing information when the evidence is ambiguous (or weak) rather than when it is strong [1]. One reason for this is that bias and evidence are weighted in the mind of the decision maker, and as the strength of the evidence increases, the effects that bias has on the decision outcome are decreased [10, 11]. Further, the more ambiguous the decision environment is, the less information the decision maker has to inform their decision, causing them to lean on prior knowledge and/or task‐irrelevant information to reach their decision. Ambiguity is therefore likely to allow the effects of bias to ferment.

Second, expertise may also lead decision makers to use top‐down processes, causing them to utilize their experience and knowledge when making decisions [12] rather than evaluating all the available information. This may cause decision makers to be accurate in simple, typical decisions, but reach wrong decisions in more complex and less typical decision environments [12]. This is because features which may help them to reach decisions in simple decision environments may not be beneficial—or may be missing—in more complex decision environments [12]. For example, the cognitive processes a super‐recognizer (i.e., those with superior facial recognition abilities [13]) uses to make a facial recognition decision in an experiment, with a non‐ambiguous face which is well lit, where they are provided with objective feedback on the outcome of their decision, may not help them if they witness a crime in a dark alley after too many glasses of wine. A second salient example can be seen where super‐recognizers may be able to use facial structures within their own ethnic group to recognize individuals but may be less effective when making recognition decisions about faces from a different ethnic background [14, 15]. In summary then, it is likely that ambiguity and expertise (represented in this study by super‐recognition ability)—alongside other factors such as time pressures (as we will discuss later [12])—may play key roles in influencing decision makers when faced with making decisions regarding facial recognition.

Techniques like linear sequential unmasking (LSU) help minimize the potential for contextual information to have a negative effect on the decision making of forensic analysts across a large array of specialisms. In the most fundamental application, when applying LSU, the examiner has no access to reference material (at the begginning of the process) from the accused or parties of interest and can only state unique features of the forensic evidence available to them. Only after stating the unique features without access to reference can examiners access the reference material and document any changes to their original analyses informed by this new information. LSU procedures can be further refined by organizing task‐relevant information by some measure of its objectivity and relevance.

### 1.2 Forensic face recognition decisions

Another factor commonly associated with false incarcerations is mistaken eyewitness identification. For example, it is estimated that mistaken eyewitness identification is present in 68% of false incarcerations [16]. Unlike decisions made by forensic practitioners, a great deal of research has been conducted on mistaken eyewitness identifications and their contributing factors (see [17]). This research has become increasingly sophisticated due to advances in neuroscience, cognitive psychology, and individual differences [18]. Research has for some time focused not solely on the face recognition abilities of the “average individual” but also on how face recognition varies from one individual to the next [19]. On one end of the spectrum, individuals exist who have very poor face recognition abilities, such as those with prosopagnosia (i.e., “face blindness”) [19]. At the other end are individuals with superior skills in the recognition, matching, and perception of faces; yet, they perform at a typical level when recognizing non‐face objects [19]. The latter have been referred to as “super‐recognizers” (SRs [20]).

The superior face perception skills of SRs suggest that they may be the ideal candidates to work as closed circuit television (CCTV) analysts [19]. However, due to the paradoxical nature of expertise [12], SRs may utilize top‐down decision‐making when making decisions on facial recognition. This means it is likely that the decisions made by SRs will be influenced/biased by their prior experience and knowledge, which may hamper performance [21].

SRs have been shown to outperform control participants on several tests, such as the Cambridge Face Perception Test (CFMT) and Cambridge Face Memory Test, as well as a before‐they‐were‐famous test [20]. For example, Robertson et al. [22] found that four SRs working for the London Metropolitan Police Service (MPS) were at an above‐average level (compared to control groups drawn from police trainees and undergraduate students) when conducting tests on both familiar and unfamiliar face matching in images of varying quality. Likewise, Davis, Forrest, Treml, and Jansari [13] found that MPS experts who were employed within the “super‐recognizer unit” (SRU) outperformed a control group at finding individuals in the Spot the Face in a Crowd Test. The MPS SRU was established in 2014 to best apply the skills of officers with superior face recognition abilities to relevant police tasks such as CCTV identification, an initiative, which has been replicated in other forces in the United Kingdom and beyond (e.g., Thames Valley, Queensland).

However, research has found critical differences between treating super‐recognizers as a group (in other words deriving the mean performance across a number of super‐recognizers) and the performance of individual super‐recognizers within that group. For one thing, as well as demonstrating that SRs performed more accurately as a group compared to controls, Robertson et al. [22] found that of the four SRs tested, none exceeded the two SD increase suggested as the threshold for SRs, that there was considerable variation between the SRs, and that all were outperformed by at least one control participant (i.e., an individual with average face perception abilities). This result was replicated by Bobak et al. [18], who tested seven SRs, finding that although they performed more accurately at the group level, not all of the individual SRs performed more accurately than the controls on the two tests of face recognition that were used. Critically, the response bias of individual SRs was as inconsistent across match and non‐matched trials as that of the control group.

As a result of this variation in the individual performance of SRs, Bate et al. [15] found that only three out of 30 police officers who had already been pre‐screened as an SR demonstrated consistently more accurate face‐matching expertise across three difficult face‐matching tests. This means that the number of individuals who could demonstrably be classified as a true expert in face recognition is low even within cohorts of SRs [23].

The above research demonstrates that it is not possible to assume superior face recognition skills within a group of individuals previously classified as SRs. Likewise, it is unwise to assume that participants recruited to be in a control group will possess either a similar face recognition ability to one another or have an ability that is lower than that of an SR. Instead, it is necessary to measure the face recognition ability of all participants within an experiment and to use this measure to designate them as being of either average or above average ability. To this end, the Cambridge Face Memory Test Long (CFMT+), a test used routinely in research exploring SRs (e.g., [19, 24]), was used in the current research to ascertain the face recognition ability of the participants.

**FIGURE 1:**
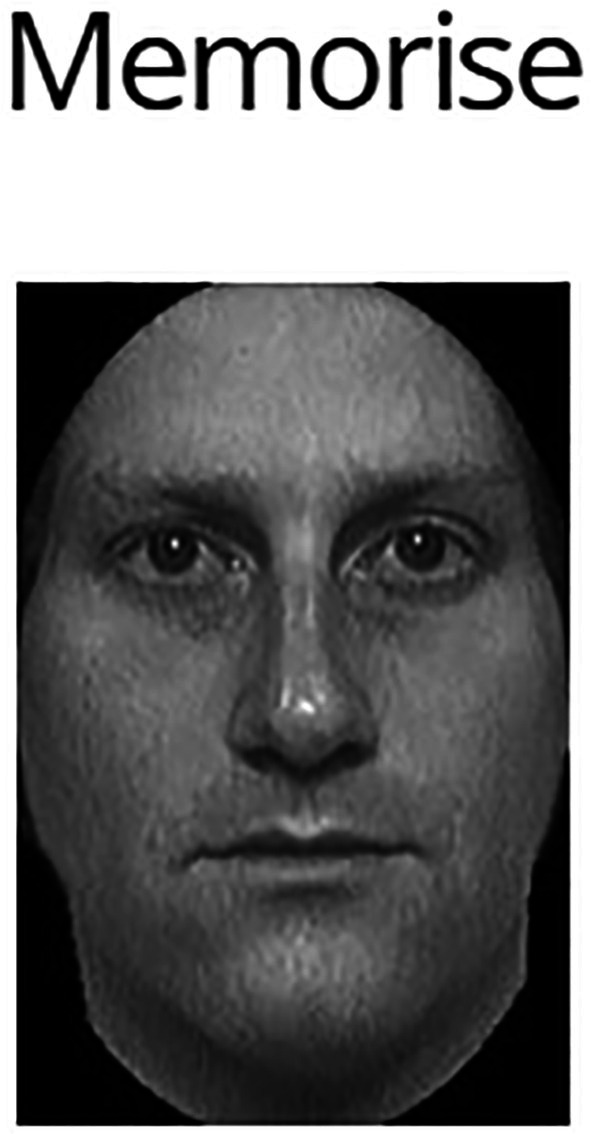
Example face from phase one of the CFMT+ study section. This figure reprinted with permission from: Russell et al. [20].

**FIGURE 2:**
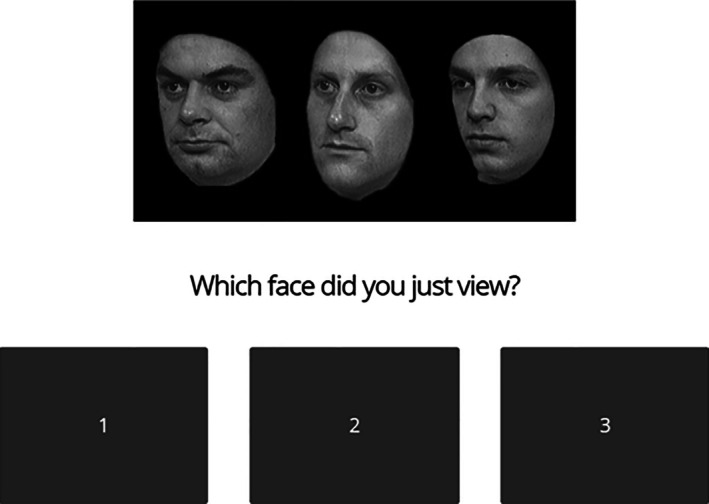
Example of response options and associated faces from phase one of the CFMT+ study section. This figure reprinted with permission from: Russell et al. [20].

**FIGURE 3:**
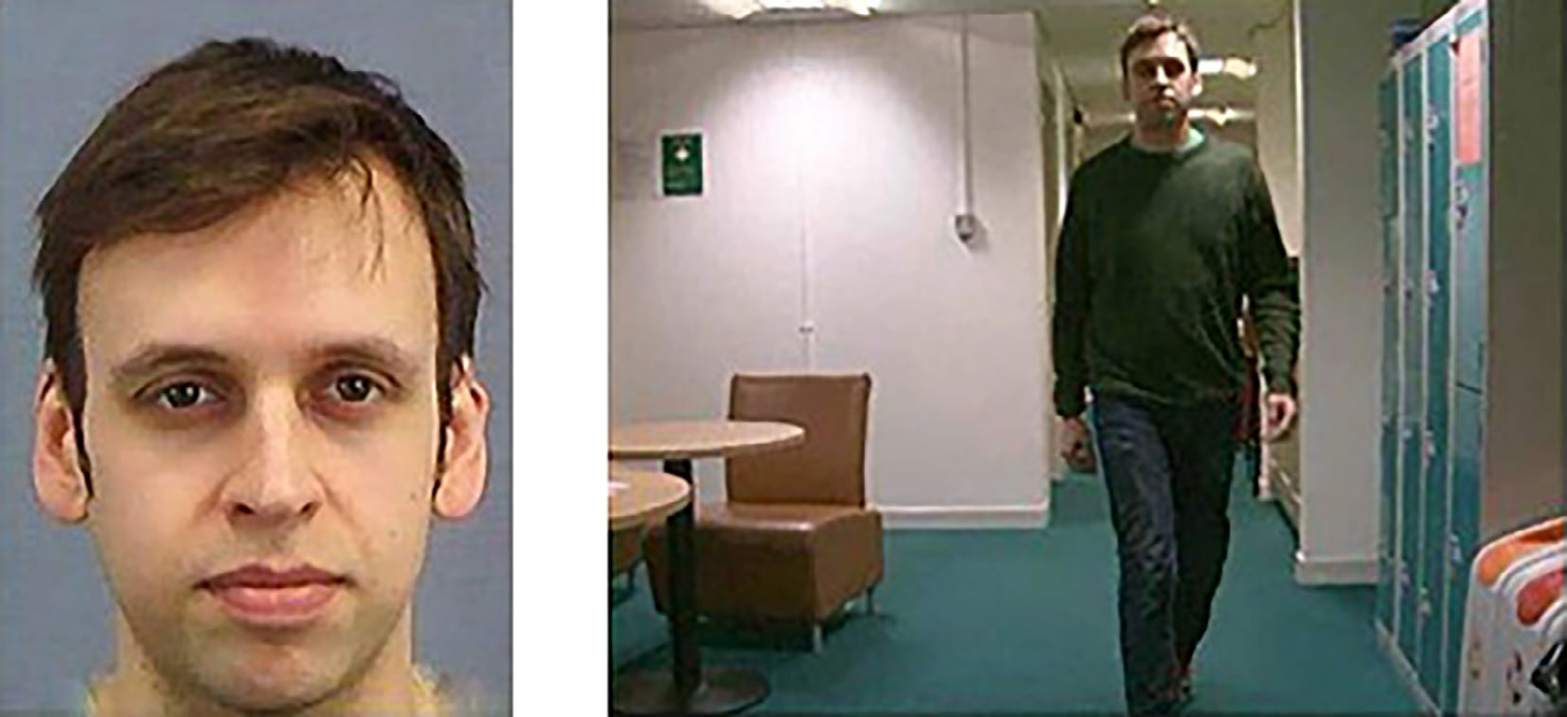
Example stimuli from CCTV video (640 × 480 pixel resolution; right) and from picture used in the corresponding face array in the face memory task (left). The target is present in the video clip on the right. This figure reprinted under the terms of the Creative Commons Attribution License from: Bobak et al. [43].

In addition, police agencies, like many institutions around the world, feel pressure to solve cases and identify dangerous individuals in order to prevent the public from harm [25]. This pressure may consciously or unconsciously spread to SRs, which may make their decisions vulnerable to the effects of bias [12]. Further, external information presented to them by their employer (e.g., severity of the crime, emotionality of the crime, knowledge of previous crimes, or the awareness of the previous decisions made by other experts/super‐recognizers) [9, 26] may also bias their decision making in relation to facial recognition—in a comparable fashion to how similar pieces of information have been shown to bias forensic scientists when making decisions [1, 2, 3]. Despite the potential for bias to influence the decisions of these facial recognition experts, no research (as far as the researchers are aware) has yet been conducted (Figures 1, 2, 3).

In addition, super‐recognizers are not the only individuals who may have to make decisions regarding facial recognition in forensic settings. The international Facial Identification Scientific Working Group distinguishes between two broad categories of face matching professionals: facial examiners, who are specialists commonly working for policing and government agencies and are called on to provide expert evidence; and facial reviewers, who are a more diverse and diversely trained group that includes people working for border control and passport agencies as well as in law enforcement [27]. Worryingly, the training that facial reviewers and examiners tend to receive can be ineffective. For example, White et al. [28] analyzed 12 published studies and found that the training received by facial reviewers led to no overall improvement in the accuracy of face matching decisions compared to control groups, and actually led to lower accuracy in half the studies. There are also considerable differences in the training provided, with few courses utilizing anything like what would be considered a full array of evidence‐based techniques [27].

The poor impact of this training is particularly concerning because, contrary to common belief, individuals are not very good at recognizing relatively unfamiliar people [29], a fact that was established by researchers some time ago [30]. This is particularly true in ambiguous settings, for example, when lighting and viewpoint change [31]. The recognition of faces from CCTV is particularly hard, as footage may not provide a clear view of the face, may only present the face for a short time period and/or be of poor quality [32]. Davis and Valentine [32], for example, found that when matching CCTV footage to a suspect in the room, 22% missed the match and 17% matched the wrong person. These difficulties (both cognitive and technological) lead to ambiguous decision environments, which not only decrease accuracy in facial recognition, but also increase the chances of bias influencing the decision [1]. Despite this, limited research has been conducted on the effects that bias may play in relation to how individuals face recognition decisions.

It is also the case that not all decisions about facial identity made within a forensic or policing context are made in isolation by an individual, but instead some involve input from multiple individuals. Research has found that working in pairs can lead to improved performance (something reported in the forensic literature as a beneficial bias; see [7]) compared to individuals [33], with the poorer performing person adopting the decision of the higher performing person, but also resulting in both partners' performance improving when the task was harder. This difference in results when using easier and harder stimuli could, as noted by the authors, simply have arisen by the use of harder stimuli avoiding the ceiling effects which may have dampened the results obtained from the easier stimuli. Whatever the explanation, it would seem prudent for future research to include stimuli that vary in difficulty, particularly that make facial recognition or matching more difficult; a manipulation that was included in the research reported here.

Across a number of experiments, Dowsett and Burton [33] found an advantage for working in pairs when making face‐matching decisions and suggest that one explanation of their results could be that poorer performers were yielding to and learning from higher performers. However, as the performance of the participants in the pairs was unknown, except implicitly, to the pairs, one interesting question is how the perception of the ability of the other participant affected decisions? In other words, if a participant has reason to believe that a decision has been made by someone with superior face‐recognition abilities, will they yield to this decision even if their own instincts suggest the opposite? This question was the focus of the current research in which the face‐recognition ability of the “other participant” was established by telling participants that they were a SR.

Dowsett and Burton [33] also note that the advantage of working in pairs could be due to social pressures, which they controlled for by manipulating whether an experimenter was present or not across two conditions in which participants worked as individuals. Again, this was explored in the current research by effectively removing the other person from the pair while still informing the participant of their decision. Furthermore, there is evidence that automatic face recognition (AFR) algorithms can bias decisions for face matching tasks, where participants have to decide whether two face images are the same person or two different people [34]. This research found that when face pairs were presented with fictitious responses from AFR, “same,” “different,” or “unresolved,” this influenced participants responses, even though they had been asked to disregard the information. Howard et al.'s [35] research also found that fictitious responses from another human could also bias face matching decisions, using this same paradigm. Both of these studies demonstrate that contextual information can bias face matching decisions; however, neither study examined the face recognition ability of those making the face matching decisions, or if this effect would transfer to a face memory recognition decision task.

### 1.3 Current study

Expanding on the work of Fysh and Bindemann [34] and Howard et al. [35], this research aims to investigate if contextual bias also influences face recognition decisions. Further, CCTV footage, like latent fingerprints, has the potential to be ambiguous (i.e., degraded CCTV images [22]). Mix this with the subjective nature of decision‐making based upon perceptual markers (nose, ears, eyes) and it is likely that bias will influence decision making, regardless of the facial expertise (super vs. non‐super‐recognizer).

The aims of the current study are threefold: (1) to establish whether decision making related to facial recognition can be biased by contextual information; (2) to assess whether or not superior facial recognition skills attenuate the influence of contextual information on face recognition; (3) to investigate if the effects of bias are attenuated in high‐quality evidence conditions when compared to low‐quality evidence conditions.

## 2 MATERIALS AND METHODS

### 2.1 Design

The study employed a 3 × 2 × 2 mixed factor experimental design; Bias (positive bias vs. negative bias vs. no bias) and target presence (absent vs. present) were within group, and evidence strength (weak video evidence vs. strong video evidence) was the between group factor. Target presence was utilized as the accuracy of facial recognition can differ depending on whether the target is present or not, with accuracy decreasing in target absent conditions [36, 37]. Three dependent measures were utilized: (1) decision confidence; (2) decision accuracy; (3) decision response time. These measures will be further explained in the materials.

Ethical approval was gained from the relevant institutional body prior to commencing the research.

### 2.2 Participants

Participants were recruited using Prolific (www.prolific.co), which is a web‐based platform that supports researchers in recruiting participants online. They were randomly allocated to either the strong or weak video evidence condition.

There were 195 participants (gender: 124 men, 68 women, two non‐binary individuals, one person who preferred not to say; sex: 125 males, 68 females, 2 prefer not to say) recruited for the study. The average age of the participants was 26.86 (*SD* = 9.42). In relation to ethnicity, the following demographics were recorded: 36 White British; 128 White other; one White Irish; five Black African; six identified as other mixed background; four preferred not to say; two Asian Indian; three from “any other Asian background”; two mixed white and Asian; one mixed White and Black Caribbean; two Black Caribbean; one Asian Pakistani; three Chinese; one other Black background.

### 2.3 Materials

#### 2.3.1 CFMT+

The CFMT+ (an addition to the CFMT) was utilized to measure the facial recognition abilities of our participants. In the initial learning phase (18 trials) of the CFMT, participants are shown six target faces (from three various angles) and then given three recognition tests for each face [38]. In the second block (30 trials), individuals are tested in their recognition of the initial six faces in new images of the faces from different perceptual angles [20]. In the third block (24 trials), participants are once again tested in their ability to recognize the targets in new images of their faces from various viewpoints, with extra visual noise added [20]. The CFMT+ extends the original test by providing an additional block (30 trials) where the images are degraded and vary regarding the amount of information available, emotionality, and facial poses. Maximum performance on the CFMT+ is 102, with super‐recognizer groups having a mean score of 95 in previous research [19, 20]. As only one participant in the current study could be categorized as a super‐recognizer under this definition, the CFMT+ score was used as a covariate, with higher scores denoting greater facial recognition abilities and lower scores highlighting poorer facial recognition abilities. Russell et al. 20 shared the CMFT+ with the research team.

#### 2.3.2 Face memory task

The face memory task required participants to view a CCTV video of a person walking along a corridor (lasting approximately 5 s). In the real world, CCTV technology develops quickly, and each individual setup will have different limitations (positioning, lighting, affordability of products with different resolutions, and features such as motion‐tracking). There are many possible ways to adjust image “quality” to reflect real‐world scenarios. In the current study, participants were randomly allocated into either “strong video evidence” (resolution of 640 × 480 pixels) or “weak video evidence” (resolution 192 × 144 pixels) group; the same videos and images were used here, only the quality of the videos varied. While face recognition abilities have been shown to be tolerant to distortions of still‐images, they seem to be significantly influenced by distortions of video footage; though interestingly, face recognition experts may have some resilience to poor‐quality images [39]. Identification of unfamiliar people from poor‐quality CCTV footage is difficult [40] and pixelation (i.e., lower resolutions) has been shown to degrade and eventually prevent face recognition [41, 42]. The original videos and the images (discussed later) were created by Bobak et al. [43], and were shared with the current research team.

Once the video had played in full, a bias statement was shown on the screen for 5 seconds. This bias statement was randomized to be either a positive bias (A super‐recognizer has said that the following image does match the face in the video), a negative bias (A super‐recognizer has said that the following image does not match the face in the video), or no bias (A super‐recognizer has not seen this video or the following image). Specifically, participants were told that either a super‐recognizer (an individual with exceptionally good face recognition abilities) had seen the video before them and they were told their decision, or that they would be the first person to see the video and be provided with no information from a super‐recognizer.

There were 36 experimental trials in total, meaning that participants were shown 12 positive bias statements, 12 negative bias statements and 12 no bias statements. Four additional trials were also shown as attention checkers, where participants were asked to give specific responses (for example, ‘please respond yes and very unconfident’). If participants failed more than two of these, they were rejected from the study due to a lack of attention (*N* = 23 participants were rejected due to failing attention checks).

After the bias statement, participants were then presented with an image of a face and asked if the face shown matched the one seen in the previous video. This decision was not time limited, so that decision response time could be measured. All target faces in these videos (mentioned earlier) and images were Caucasian males to control for gender and racial perceptual biases [18]. The images of faces were cropped using photo editing software to remove distracting clothing and/or jewelry [18].

In half of the trials, the target was present, meaning that the same person from the video was in the photograph. In the remaining half, the targets were absent, meaning that the test photograph consisted of a similar person who had not been seen before. Piloting was conducted to ensure that the target absent person looked similar to the person in the video, and face validity was ensured by getting agreement from the authors of the study.

It should also be mentioned that careful counterbalancing was conducted to ensure that each video/face was in each of the within‐subject's conditions (e.g., (1) target absent and positive bias; (2) target absent and negative bias; (3) target absent and no bias; (4) target present and positive bias; (5) target present and negative bias; (6) target present and no bias) over the set of 195 participants. This was done by creating six variations of the experiment, where each of the 36 videos/faces was placed in a different condition for each of the six sets of participants. Participants were randomly allocated to each of these six variations. This was done to ensure effects were caused by each of the within‐subject factors rather than due to individual faces. This happened for both the weak and strong evidence conditions.

Following each response, a 5‐point Likert scale was shown, asking participants to indicate how confident they were in their decision from one (not at all confident) to five (very confident).

### 2.4 Procedure

This study was created and hosted on the Gorilla Experiment Builder (www.gorilla.sc) [44], a cloud‐based research platform. Following recruitment and obtainment of informed consent, the face matching task was given. Next, participants completed the CFMT+ and a demographic questionnaire. Upon experiment completion, a full debrief was then presented on screen, and participants were directed back to Prolific to receive payment for their time (approximately £7.56 per hour).

## 3 RESULTS

### 3.1 Layout of results

Descriptive statistics are presented for each of the covariate, different factors, and measures in three tables. Correlational analysis is conducted to test if associations exist between the CFMT+ scores and the overall accuracy, confidence, and decision times of the participants. These variables, minus the CFMT+, are then correlated with each other after the data are separated into trials that were congruent with the SR statement and trials that were incongruent with the SR statement. Each of the dependent measures (i.e., decision confidence, decision accuracy, and decision response time) is then analyzed across each of the factors (bias, target presence, evidence strength) using a generalized estimated equation (GEE).

### 3.2 Descriptive statistics

All analyses are calculated using all trials in each condition, including both incorrect and correct responses.

Tables 1 and 2 present the mean and standard deviations for the total accuracy and total confidence scores for each of the iterations of target presence, bias, and evidence strength. Table 3 presents the mean and standard deviation for the average decision time score across each of the factor iterations.

**TABLE 1:** Mean and standard deviations for the total accuracy scores by condition. Conditions in which the bias was congruent with target presence (e.g., says was present and was present) are in bold. Each condition had six repetitions or trials for a maximum possible score of 6.00.

Target	Bias	Evidence strength	Accuracy total (SD)
**Absent**	**Negative**	**High**	**5.19 (0.83)**
Absent	Positive	High	4.81 (1.17)
Absent	No bias	High	4.83 (1.11)
Present	Negative	High	4.66 (1.22)
***Present***	***Positive***	***High***	***5.01 (1.09)***
*Present*	*No bias*	*High*	*5.02 (1.02)*
**Absent**	**Negative**	**Low**	**4.82 (1.21)**
Absent	Positive	Low	4.29 (1.41)
Absent	No bias	Low	4.61 (1.15)
Present	Negative	Low	4.40 (1.41)
**Present**	**Positive**	**Low**	**5.01 (1.06)**
Present	No bias	Low	4.87 (1.11)

**TABLE 2:** Mean and standard deviation total confidence scores by condition. Conditions in which the bias was congruent with target presence are in bold. Each confidence score was rated on a 5‐point scale for six trials, for a maximum possible confidence total of 30.

Target	Bias	Evidence strength	Confidence total (SD)
**Absent**	**Negative**	**High**	**24.20 (3.52)**
Absent	Positive	High	23.18 (3.79)
Absent	No bias	High	23.92 (3.55)
Present	Negative	High	22.73 (3.49)
**Present**	**Positive**	**High**	**23.45 (3.52)**
Present	No bias	High	22.93 (3.11)
**Absent**	**Negative**	**Low**	**23.53 (3.81)**
Absent	Positive	Low	22.85 (3.76)
Absent	No bias	Low	22.65 (3.36)
Present	Negative	Low	22.01 (3.60)
**Present**	**Positive**	**Low**	**23.57 (3.57)**
Present	No bias	Low	23.34 (3.46)

**TABLE 3:** Mean and standard deviation for the average decision times (milliseconds) by condition. Conditions in which the bias was congruent with target presence are in bold.

Target	Bias	Evidence strength	Decision time mean (SD)
**Absent**	**Negative**	**High**	**3274.72 (2525.56)**
Absent	Positive	High	3562.35 (4210.63)
Absent	No bias	High	2872.48 (1378.61)
Present	Negative	High	3571.15 (3057.40)
**Present**	**Positive**	**High**	**2893.95 (1817.12)**
Present	No bias	High	2810.56 (1178.73)
**Absent**	**Negative**	**Low**	**3297.97 (1490.67)**
Absent	Positive	Low	3339.73 (1908.75)
Absent	No bias	Low	2995.24 (1129.54)
Present	Negative	Low	4192.19 (6327.96)
**Present**	**Positive**	**Low**	**2760.20 (1229.05)**
Present	No bias	Low	2988.23 (1638.04)

Interestingly, accuracy scores were seen to be mostly higher when the bias condition was congruent with the target presence condition. The only exception to this was when the target was present and the evidence strength was high; here, the accuracy score did not differ much in the no bias condition from the positive bias condition (in italics) (Table 1).

Similarly to the accuracy scores, it seems the confidence scores were higher when the bias condition was congruent with the target presence condition (Table 2).

The average decision time was quicker when the bias condition was congruent with the target presence condition, when looking at the positive and negative bias conditions. However, it seems that decision‐makers were quicker when making decisions in the no bias condition when compared to the other two conditions (Table 3).

The final measure used in the current study was the CFMT+ score (*M* = 67.24; *SD* = 12.16; variance = 147.76); due to only one participant scoring above the cut‐off for recognition as a super‐recognizer, this score was utilized as a covariate. The minimum score was 40 and the maximum was 96. This variable was normally distributed, with most scores around the average, and fewer scores at the extremes (e.g., poor and superior facial recognition).

### 3.3 Investigating the relationships between CFMT+ scores and dependent measures

To test the association that the CFMT+ score had with total accuracy, total confidence, and average decision time, three correlations were conducted; total here refers to all the trials over the whole experiment, rather than particular blocks or conditions. None of the correlations were significant. Due to this, the CFMT+ was dropped as a covariate from further analyses.

Additional correlations were also conducted between the three dependent variables (accuracy, decision time and confidence) after separating out data in which the target was present from data in which the target was absent. In relation to the target present condition, accuracy was found to significantly correlate with confidence [*r*(195) = 0.354, *p* = <0.001] and decision time [*r*_*s*_(195) = −0.213, *p* = 0.003]. Decision time was also significantly and negatively correlated with confidence [*r*_*s*_(195) = −0.265, *p* = <0.001]. None of the outcome variables were significantly correlated with the CFMT+ scores.

In relation to the target absent condition, accuracy was found to significantly correlate with confidence [*r*(195) = 0.272, *p* = <0.001] and decision time [*r*_*s*_(195) = −0.178, *p* = 0.013]. Decision time was also significantly correlated with confidence [*r*_*s*_(195) = −0.322, *p* = <0.001]. It seems over both target presence conditions, the more confident the decision maker was, the more accurate they were, and the quicker their decision was made, the more confident and accurate they were (see Appendix S1 for more details).

### 3.4 Investigation of the effect of bias, target presence, evidence strength, and facial recognition ability (CFMT+) on decision making

#### 3.4.1 Accuracy

In relation to the accuracy data, Shapiro–Wilk's tests highlighted that the said measure was not normally distributed across each of the factors. Therefore, a generalized estimating equation (GEE) was used. GEEs allow data that do not meet parametric assumptions and that have been collected from a longitudinal study, clustered data, or a repeated measures design to be analyzed [45]. The dependent variable in the model was accuracy; the predictors were bias, target presence, and evidence strength; and the CFMT+ score acted as a covariate.

First, the most suitable correlation matrix, link function, and distribution were tested using the quasi‐likelihood under independence model criterion in the goodness of fit box (QIC). An autoregressive correlation matrix, with a negative binomial distribution and negative binomial link function, was chosen as it led to the most parsimonious model (QIC = 61.064) when compared to combinations of other matrices, other relevant distributions (e.g., Poisson as accuracy scores were count data) and link functions. However, it should be mentioned that the inverse Gaussian distribution led to a better fit for the data, but was not chosen as said distribution did not include zeros within the analysis, which would not be optimal here as accuracy data were being analyzed.

First, the intercept was found to be significant [*X* ^2^ (1) = 488.704, *p* < 0.001]. Second, bias was found not to be a significant predictor of accuracy [*X* ^2^ (2) = 1.458, *p* = 0.48]. Third, target presence was found not to be a significant predictor of accuracy [*X* ^2^ (1) = 0.577, *p* = 0.45]. Fourth, evidence strength was found to be a significant predictor of accuracy [*X* ^2^ (1) = 11.415, *p* = 0.001]. Individuals in the high‐quality evidence group (Estimated Marginal Means; *EMM* = 4.91) were significantly more accurate than individuals in the low‐quality evidence group (*EMM* = 4.65).

Evidence strength and bias [*X* ^2^ (2) = 1.099, *p* = 0.58], evidence strength and target presence [*X* ^2^ (1) = 1.395, *p* = 0.24], and evidence strength, target presence, and bias [*X* ^2^ (2) = 3.388, *p* = 0.18] were found not to be significant interactions.

There was a significant interaction for bias and target presence [*X* ^2^ (2) = 35.174, *p* < 0.001]. Bonferroni post hoc tests revealed the following. First, accuracy was significantly (*p* = 0.007) higher when the bias was negative and the target was absent (*EMM* = 5.003) than when the bias was negative and the target was present (*EMM* = 4.53). Second, accuracy was significantly (*p* = 0.004) lower when the bias was positive and the target was absent (*EMM* = 4.53) than when the bias was positive and the target was present (*EMM* = 5.01). Third, there was no significant difference in accuracy (*p* = 0.95) when no bias was present and the target was absent (*EMM* = 4.72) when compared to when no bias was present and the target was present (*EMM* = 4.94).

In addition, in the target absent condition, it was found that accuracy was significantly higher when a negative bias was presented than when a positive bias (*p* < 0.001) or no bias was presented (*p* = 0.03). No significant differences existed here when comparing positive bias with when no bias is present (*p* > 0.999). In the target present condition, it was found that accuracy was significantly higher when a positive bias was presented than when a negative bias (*p* < 0.001) was shown, but did not significantly differ when no bias was presented (*p >* 0.999). A significant difference existed here when comparing the negative bias with when no bias was present (*p* < 0.001), with neutral being higher than negative. The results above were confirmed by a separate 2 × 2 × 3 mixed analysis of variance (ANOVA).

Finally, the covariate (CFMT+) was found not to be a significant predictor of accuracy [*X* ^2^ (1) = 0.239, *p* = 0.63], and its removal improved the model fit (previous QICC = 86.134 vs. current QICC = 84.142) and did not influence any existing main effects or interactions. Follow‐up Spearman's correlations highlighted that the CFMT+ score correlated with accuracy when participants were presented with no bias and the target was present [*r*_*s*_ (195) = −0.15, *p* = 0.02]; this was not found when other iterations of bias and target presence were presented to participants. Interestingly, this highlights that individuals with superior face recognition abilities did not outperform individuals lower in the scale when bias was present or when the target was absent, and actually performed worse in the no bias condition when the target was present.

#### 3.4.2 Confidence

In relation to the confidence data, Shapiro–Wilk's tests highlighted that the measure was not normally distributed across some of the factors. Therefore, a GEE was used. An auto‐regressive correlation matrix, with an inverse gaussian distribution and a log link function, was used as it led to the most parsimonious model (QIC = 27.188) when compared to combinations of other matrices, other relevant distributions (e.g., Tweedie, Gaussian, gamma) and link functions. The same factors, covariate, and dependent variable were used here as in the model above.

First, the intercept was found to be significant [*X* ^2^ (1) = 5516.804, *p* < 0.001]. Second, bias was found not to be a significant predictor of confidence [*X* ^2^ (2) = 0.869, *p* = 0.65]. Third, target presence was found not to be a significant predictor of confidence [*X* ^2^ (1) = 2.903, *p* = 0.09]. Fourth, evidence strength was found not to be a significant predictor of confidence [*X* ^2^ (1) = 1.307, *p* = 0.25].

Evidence strength and bias [*X* ^2^ (2) = 3.144, *p* = 0.21], and evidence strength and target presence [*X* ^2^ (1) = 2.265, *p* = 0.13] were found not to be significant interactions. Two significant interactions were found, however.

First, bias and target presence were found to share a significant interaction [*X* ^2^ (2) = 29.663, *p* < 0.001]. Bonferroni post hoc tests were used to further investigate this interaction. When the target was absent, a negative bias (*EMM* = 23.86) led to significantly (*p* = 0.02) higher confidence scores than a positive (*EMM* = 23.01), but did not differ significantly (*p* = 0.27) from no bias (*EMM* = 23.28). When the target was absent, a positive bias did not differ significantly (*p* > 0.999) from when no bias was presented. When the target was present, a negative bias (*EMM* = 22.37) led to significantly lower confidence scores than a positive bias (*p* < 0.001; *EMM* = 23.51) and no bias (*p* = 0.04; *EMM* = 23.13). No significant difference (*p* > 0.999) existed between when no bias was present and when a positive bias was present, when the target was present.

When a negative bias was present, confidence was significantly (*p* < 0.001) higher when the target was absent than when it was present. When a positive bias was present, confidence was not significantly (*p >* 0.999) different when the target was absent than when it was present. Similarly, confidence ratings did not differ significantly (*p > 0*.999) across target absent and present when no bias was given.

Second, bias, target presence, and evidence strength were found to share a significant interaction [*X* ^2^ (2) = 8.042, *p* = 0.02]. As there are a number of interactions within each of these three factors, only significant interactions (using Bonferroni correction) will be reported (see Appendix S2 for more details):

- First, when the evidence strength was high and bias negative, confidence ratings were significantly (*p* = 0.02) higher when the target was absent (*EMM* = 24.20) than when the target was present (*EMM* = 22.73).
- Second, when the evidence strength was high, the target was absent, and the bias was negative, confidence ratings were significantly (*p* = 0.001) higher than when the evidence strength was low, the target was present, and the bias was negative (*EMM* = 22.02).
- Third, when the evidence strength was high, the target was absent, and there was no bias (*EMM* = 23.91), confidence scores were significantly (*p* = 0.01) higher than when the evidence strength was low, there was a negative bias, and the target was present.
- Fourth, when the evidence strength was low and the target was present, confidence scores were significantly (*p* = <0.001) lower when the bias was negative than when the bias was positive (*EMM* = 23.57).
- Fifth, when the evidence strength was low and the target was present, confidence scores were significantly (*p* = 0.02) higher when there was no bias (*EMM* = 23.34) than when the bias was negative.

Finally, the covariate (CFMT+) was found not to be a significant predictor of confidence [*X* ^2^ (1) = 1.295, *p* = 0.26], and its removal improved the model fit (previous QICC = 27.33 vs. current QICC = 25.34) and did not influence any existing main effects or interactions. Follow‐up Spearman's correlations highlighted that the CFMT+ score did not significantly correlate with confidence in any of the bias and target presence iterations.

#### 3.4.3 Decision time

In relation to the decision time data, Shapiro–Wilk's tests highlighted that the said measure was not normally distributed across some of the factors and the data had a large positive skew (12.06). Therefore, a GEE was used. An inverse Gaussian distribution, with an exchange correlation matrix and log link function, was used as it led to the most parsimonious model (QIC = 71.603) when compared to combinations of other matrices and other relevant distributions (e.g., Tweedie, Gaussian, gamma). However, it should be mentioned that an identity link function did lead to a more parsimonious model (QIC = 69.048) but did not allow for main effects to be analyzed; thus, the log link function was chosen. The same factors and dependent variable were used here as in the model above.

First, the intercept was found to be significant [*X* ^2^ (1) = 1775.407, *p* < 0.001]. Second, bias was found to be a significant predictor of decision times [*X* ^2^ (2) = 18.694, *p* < 0.001]. Post hoc Bonferroni tests were utilized to explain this finding. Negative bias (*EMM* = 3565.85) led to significantly slower decision times than when a positive bias was presented (*p* = 0.01; *EMM* = 3123.42) and when no bias was presented (*p* < 0.001; *EMM* = 2914.92). Further, no significant difference (*p* = 0.16) existed in decision times when comparing a positive bias with no bias. Third, target presence was found not to be a significant predictor of decision times [*X* ^2^ (1) = 0.271, *p* = 0.60]. Fourth, evidence strength was found not to be a significant predictor of decision times [*X* ^2^ (1) = 0.106, *p* = 0.75].

Evidence strength and bias [*X* ^2^ (2) = 3.774, *p* = 0.15], evidence strength and target presence [*X* ^2^ (1) = 1.007, *p* = 0.32], and evidence strength, target presence, and bias [*X* ^2^ (2) = 0.620, *p* = 0.73] were found not to share significant interactions. The only interaction that was found to be significant existed in relation to bias and target presence [*X* ^2^ (2) = 17.219, *p* < 0.001]. Bonferroni post hoc tests were used to expand upon these results.

When a negative bias was presented, decision times were quicker when the target was absent (*EMM* = 3287.20) when compared to when it was present (*EMM* = 3868.14), but this difference was not significant (*p* = 0.83). When a positive bias was presented, decision times were quicker when the target was present (*EMM* = 2826.13) in comparison to when it was absent (*EMM* = 3451.99); this difference was significant (*p* = 0.01). When no bias was presented, decision times were slower when the target was absent (*EMM* = 2932.62) than when they were present (*EMM* = 2897.32); this effect was not significant (*p* > 0.999).

When the target was absent, decision times did not differ significantly between when a positive bias was presented or when a negative bias was presented (*p* > 0.999), when a negative bias was presented or when no bias was presented (*p* = 0.07), or when a positive bias was presented or when no bias was presented (*p* = 0.08). When the target was present, decision times were significantly longer when a negative bias was presented than when a positive bias was presented (*p* = 0.01) or when no bias was presented (*p* = 0.03). No significant differences (*p* > 0.999) existed in decision times when comparing positive bias and no bias when the target was present.

Finally, the covariate (CFMT+) was found not to be a significant predictor of decision times [*X* ^2^ (1) =0.058, *p* = 0.81], and its removal improved the model fit (previous QICC = 26.076 vs. current QICC = 24.076) and did not influence any existing main effects or interactions. Follow‐up Spearman's correlations highlighted that the CFMT+ score did not significantly correlate with decision times in any of the bias and target presence iterations.

### 3.5 Summary of main findings

The accuracy of, and confidence in, the decisions was biased toward the decision made by the fictional SR. There was a significant interaction between the bias and the target presence factors, with accuracy and confidence increasing and decision times decreasing when the bias statement was congruent with the target presence condition. Face recognition abilities did not attenuate the impact of bias on the recognition decisions. Finally, while strong video evidence resulted in higher accuracy in general, the interaction between evidence strength and bias on accuracy was not significant.

## 4 DISCUSSION

The aims of the current study were threefold: (1) to establish whether decision‐making related to facial recognition can be biased by contextual information; (2) to assess whether or not superior facial recognition skills attenuate the influence of contextual information on face recognition; (3) to investigate if the effects of bias are attenuated in high‐quality evidence conditions when compared to low‐quality evidence conditions. Each of these aims will now be discussed.

First, the research highlighted that decisions (accuracy, confidence, and decision time) were influenced by both the contextual information (i.e., the decision of the previous, fictional super‐recognizer) and the target presence condition. There was significantly greater accuracy when the negative bias was present and the target was absent (bias congruent with correct decision) than when negative bias was present, and the target was present (bias incongruent with correct decision); this congruency effect held when the target present condition was congruent with the positive bias condition. Interestingly, no differences existed in the no bias condition regardless of whether the target was present or absent.

The interaction between target presence and bias also influenced the confidence and decision time of the decision‐maker. Therefore, the results show that when there is a congruency effect between the bias and the target presence factors, accuracy and confidence increase, and decision times decrease. (These results highlight that the decision‐making behind face recognition decisions is influenced by contextual information, replicating results for face matching studies investigating cognitive bias through fictitious AFR responses [34, 35]. This finding adds to a growing awareness of how susceptible to bias actors involved in criminal justice and related fields may be (e.g., forensic scientists [1]; police investigators [46]; legal laypeople and jurors [47]). To our knowledge, the current study is the first to demonstrate such influence of bias in face recognition, using a memory task.

The research shows that this bias may be positive when congruent with the target presence, but negative when incongruent. Previous research has similarly discussed the positives [33] and negatives [7, 8] of forensic decision makers working together. Regardless on the impact contextual information may have on the accuracy of the individual forensic decision‐maker, the effects of bias may undermine justice further along the criminal justice system [48]. Thompson [48] has discussed how contextual information may aide individual decision‐makers to reach an accurate outcome but will “undermine the independence of the … evidence” (p. 131). According to Bayesian norms, if the second facial examiner is provided with contextual information from a prior examiner, these two pieces of evidence (prior and current facial examiner) will become tied and no longer have conditional independence, as the accuracy of the second decision is dependent on the decision/opinion of the prior super‐recognizer [48]. Due to this, the probative value of the second facial examiners evidence will be decreased and increase the chance of a false positive [48].

Second, and interestingly, when the CFMT+ score was used as a covariate, it did not influence the significance of the overall model or the main effects and interactions. This highlights that superior face recognition abilities, and thus expertise, may not increase or attenuate the role that bias plays in decision‐making. This research supports the work of Dror et al. [12] who suggest that experts have no more resistance to the effects of bias than laypeople, although it also shows they may be no more vulnerable to said effects. Three explanations may explain the current finding. The first is that due to complexities and difficulties surrounding face recognition, both cognitive (individuals find the recognition of unfamiliar faces difficult; [30]) and technological (CCTV images may only present the face for a short period and show degraded images), even “face experts” are not perfect, and although they may be better at recognizing faces, their superiority in said skill is not protective against contextual and confirmation biases [12].

The other explanation is that individuals who scored above average on the CFMT+ scale are not a homogeneous group and the CFMT+ does not allow researchers to capture a unique phenomenon. Thus meaning that individuals higher up on the scale may have varying and unique cognitive abilities that help them recognize faces [24]. In other words, there may be different types of “facial experts.” For instance, some individuals with superior facial recognition abilities may recognize faces due to specific, inner face features such as nose shape, whereas others may recognize faces more holistically. Therefore, there is no such thing as one type of facial expert and thus it may be difficult to categorize them as a homogeneous group of experts. This means that some facial experts might be better than others and are *potentially* better at ignoring bias; future research should aim to test this, as the extant literature remains lacking in detail on the “types” of facial recognition experts.

The third and most likely explanation based on the current data (as accuracy did not correlate with CFMT+ score) is that individuals scoring high on the CFMT+ do not *always* have superior face recognition abilities (screening for super recognition is so far varied and should not be based on the results of a single test or self‐selection [49, 50]); and cannot *always* be deemed as experts in faces, meaning it is difficult to establish the interaction that bias and expertise may have in this domain. Future research, however, should replicate the current design with other measures, such as the beSure‐Berlin test [51]. The beSure‐Berlin test is made up of 5 sub‐tests which were created using authentic materials from the real world, allowing for the identification of those with superior facial recognition using applied tests such as static image comparison and dynamic crown search [51]. By using additional tests such as the BeSure‐Berlin test ‐ alongside the CFMT+ ‐ it may make it easier to identify genuine facial experts (i.e., those with superior facial recognition). Other factors not studied here, such as perceived ability, metacognition, and ability to resist bias, may also play a role and should be investigated in future research [52, 53].

The results of the current study highlight that the main conclusion of Dror et al. [3, 9, 54] (i.e., that contextual biases can influence decision‐making) extends to forensic decisions made using a different decision context (facial recognition) and different materials (faces rather than fingerprints and DNA evidence). Contextual bias is pervasive and can influence the judgments of decision‐makers across multiple paradigms [55]. Similarly to forensic scientists, the potential for police officers, CCTV analysts, and border control officers to be influenced by bias (i.e., contextual information regarding a prior decision) during face recognition decisions can be extremely serious [27, 28]. The wrong individuals may be charged, or worse, brought before a jury, thus increasing the chances of a miscarriage of justice occurring. This is particularly concerning in the jury context, as evidence may be perceived as conditionally independent and corroborating by the trier of fact, when in fact it has conditional dependence and is intrinsically linked [48].

One recommendation to decrease the influence that bias may play in the decision‐making of individuals who have to make decisions regarding facial recognition (e.g., CCTV analysts) [22] may be for said individuals to employ a linear sequential unmasking (LSU) technique [55]. In LSU, the amount of information that is available to forensic decision‐makers is controlled, thus attenuating the role of bias in the decision [43]. In the beginning step of this technique, forensic scientists are only shown the trace evidence (the evidence from the crime scene) before being shown the reference material (i.e., the evidence from the suspect [55]). In regard to a face recognition decision, this would mean that the facial examiner would analyze (and document) the CCTV image of the suspect before they are shown a clear image of a suspect; if the person was identified by a facial examiner and that is why they are a suspect, then an independent facial examiner should begin the LSU technique. This procedure would stop the high‐quality image of the suspect and/or knowledge of the previous decision makers decision biasing the facial examiner into seeing the suspect's face in the CCTV [43, 55]. Once the facial examiner has analyzed the trace evidence (face in this case) and documented information about the face (shape of nose, color of eyes, eye size), they will be provided with the reference material to analyze and compare [55]. The examiner is allowed a limited number of opportunities to then go back and re‐evaluate the trace evidence. Please see Dror et al [55] for more details surrounding LSU.

A technique that could then follow up this procedure would be for an independent facial examiner to then go through the same LSU procedure (again), independent of the initial decision, to establish if a similar decision can be reached. This method would stop the secondary facial examiners' decision from being biased by the primary decision.

Third, the results of the current study highlight that the effects of bias were not attenuated in high quality evidence conditions when compared to low‐quality evidence conditions, as there was no significant interaction between video quality, target presence, and bias in relation to accuracy. High‐quality evidence did lead to significantly more accurate decisions in general when compared with low quality evidence, but as mentioned above, this did not interact with other factors. The evidence quality factor may not have interacted with the bias conditions, in relation to accuracy scores, because the quality of the materials did not deviate enough to cause the effects of bias to outweigh the role of evidence within the decision‐making process [10, 11]. This can be highlighted by the fact that the mean accuracy results for the high‐ and low‐quality evidence conditions were not drastically different, despite being significant (*M* = 4.91 vs. *M* = 4.65, respectively). However, CCTV evidence is commonly grainy, meaning it is unlikely that CCTV analysts and Police officers are presented with higher quality evidence in the field (Davis & Valentine, 2008).

### 4.1 Limitations and future research

The major limitation of the current research is a lack of super‐recognizers recruited in the sample, as only one participant could be categorized as a super‐recognizer according to the established CFMT+ cut‐off [27, 56]. Future research therefore would hope to replicate the current study targeting exclusively super‐recognizers, using multiple inventories (such as CFMT+ and beSure‐Berlin test). Despite this, however, in the current analysis, the research team decided to keep the CFMT+ score as a scale variable, rather than categorizing individuals as super or not super‐recognizers, meaning that the statistical models used would have had more sensitivity to test whether or not facial recognition ability influenced the effects of bias and/or was related to measures such as accuracy and confidence. Despite this, no significant effects were found.

In addition, another major limitation of the current research may relate to the fact that the research was conducted online. Due to the Covid‐19 pandemic, participants were recruited using Prolific and tested using Gorilla while in their individual uncontrolled environments. Face recognition tasks such as the CFMT+ suffer from poor ecological validity [51] in exchange for enhanced experimental control when performed in a laboratory environment, which we could not provide. Likewise, the critique of the CFMT+ lacking realistic facial tasks could be improved upon in future research by using other inventories such as the beSure‐Berlin test [51].

Quick and significant changes in CCTV technology and availability (from grainy footage covering some shopfronts, to high‐definition motion‐tracking front‐door cameras in many domestic homes) may require adaptations to future face recognition materials to more closely match what is now commonly available. While we included resolution as our “quality” variable, there are other methods of adjusting video “quality” that could be considered alongside this new technology.

## 5 CONCLUSION

In conclusion, the results showed a congruency effect, that is, the decision confidence, accuracy, and decision time were influenced by whether the bias condition matched the target presence condition. Further, when the bias condition matched the target presence condition, the accuracy increased, the confidence increased, and decision speed decreased. However, when the bias condition did not match the target presence condition, the accuracy decreased, the confidence decreased, and the decision time increased, showing the negative effects that contextual bias can have on forensic decision‐makers. The findings helped to show that the findings of Dror et al. [9] can be generalized to a different sample of forensic decision makers, using different stimuli, in a more internally controlled setting. Interestingly, facial recognition ability did not mediate the effects of bias or correlate with key measures such as accuracy and confidence. Implications of this research are that units that employ facial experts (e.g., Thames Valley Police) should employ LSU and independent checking procedures to minimize the role that bias may play in facial recognition decisions.

## FUNDING INFORMATION

British Academy/Leverhulme Small Research Grant SRG1920\100508.

## CONFLICT OF INTEREST STATEMENT

The authors have no conflicts of interest to declare for this paper.

## Supporting information


Data S1.

## Data Availability

The data that support the findings of this study are available on request from the corresponding author. The data are not publicly available due to privacy or ethical restrictions.
