# Supplementary material for: The effects of contextual bias on face recognition decisions
Source: J Forensic Sci. 2025 Sep 9;70(6):2420–33. doi: 10.1111/1556-4029.70177 (PMC12584121; doi:10.1111/1556-4029.70177)
Supplement: Supplementary file 1 — Data S1. [file JFO-70-2420-s001.docx]

**Supplemental Material: Appendices**

APPENDIX 1 Correlations.


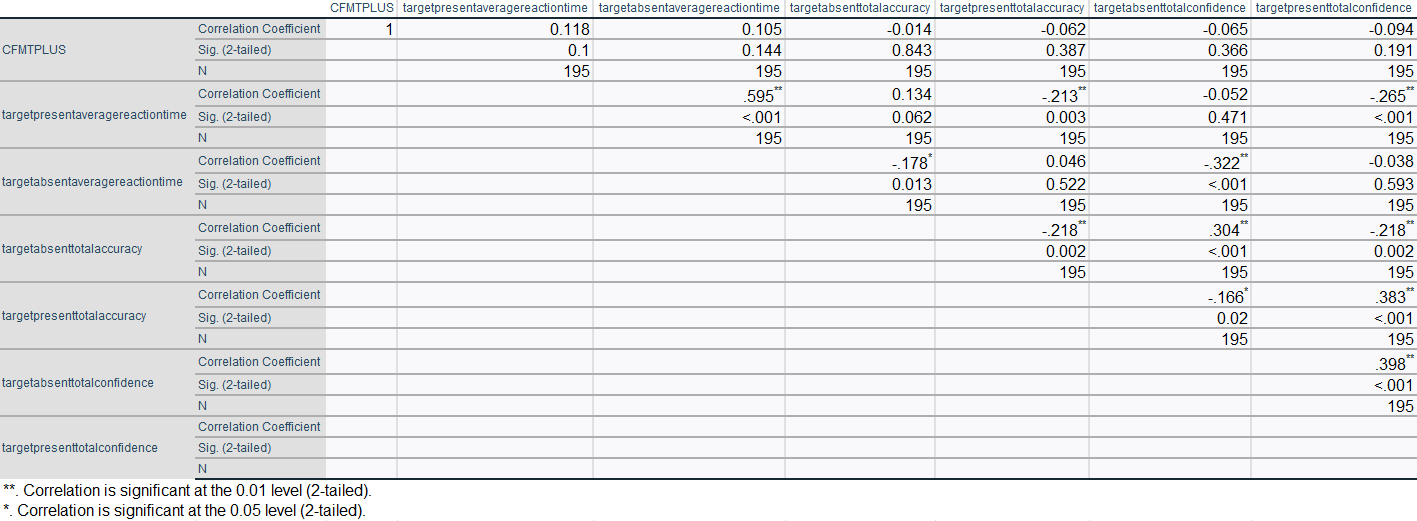


| APPENDIX 2 Pairwise comparisons. | | | | | | | |
| --- | --- | --- | --- | --- | --- | --- | --- |
| (I) VIDEOQUAL*bias*target | (J) VIDEOQUAL*bias*target | Mean Difference (I-J) | Std. Error | df | Bonferroni Sig. | 95% Wald Confidence Interval for Difference | |
|  |  |  |  |  |  | Lower | Upper |
| [VIDEOQUAL=1.00]*[bias=1.00]*[target=1.00] | [VIDEOQUAL=1.00]*[bias=1.00]*[target=2.00] | 1.4737^a^ | 0.4004 | 1 | 0.015 | 0.1253 | 2.8222 |
|  | [VIDEOQUAL=1.00]*[bias=2.00]*[target=1.00] | 1.0266 | 0.334 | 1 | 0.139 | -0.098 | 2.1514 |
|  | [VIDEOQUAL=1.00]*[bias=2.00]*[target=2.00] | 0.7547 | 0.4275 | 1 | 1 | -0.685 | 2.1943 |
|  | [VIDEOQUAL=1.00]*[bias=3.00]*[target=1.00] | 0.2889 | 0.3479 | 1 | 1 | -0.883 | 1.4606 |
|  | [VIDEOQUAL=1.00]*[bias=3.00]*[target=2.00] | 1.2777 | 0.4382 | 1 | 0.234 | -0.198 | 2.7535 |
|  | [VIDEOQUAL=2.00]*[bias=1.00]*[target=1.00] | 0.6682 | 0.5201 | 1 | 1 | -1.083 | 2.4197 |
|  | [VIDEOQUAL=2.00]*[bias=1.00]*[target=2.00] | 2.1837^a^ | 0.5056 | 1 | 0.001 | 0.4809 | 3.8866 |
|  | [VIDEOQUAL=2.00]*[bias=2.00]*[target=1.00] | 1.3432 | 0.5184 | 1 | 0.631 | -0.403 | 3.0889 |
|  | [VIDEOQUAL=2.00]*[bias=2.00]*[target=2.00] | 0.6252 | 0.5039 | 1 | 1 | -1.072 | 2.3221 |
|  | [VIDEOQUAL=2.00]*[bias=3.00]*[target=1.00] | 1.5437 | 0.4888 | 1 | 0.105 | -0.102 | 3.1898 |
|  | [VIDEOQUAL=2.00]*[bias=3.00]*[target=2.00] | 0.857 | 0.4946 | 1 | 1 | -0.809 | 2.5228 |
| [VIDEOQUAL=1.00]*[bias=1.00]*[target=2.00] | [VIDEOQUAL=1.00]*[bias=1.00]*[target=1.00] | -1.4737^a^ | 0.4004 | 1 | 0.015 | -2.822 | -0.125 |
|  | [VIDEOQUAL=1.00]*[bias=2.00]*[target=1.00] | -0.4471 | 0.4227 | 1 | 1 | -1.871 | 0.9766 |
|  | [VIDEOQUAL=1.00]*[bias=2.00]*[target=2.00] | -0.7191 | 0.3325 | 1 | 1 | -1.839 | 0.4006 |
|  | [VIDEOQUAL=1.00]*[bias=3.00]*[target=1.00] | -1.1848 | 0.4224 | 1 | 0.332 | -2.608 | 0.2378 |
|  | [VIDEOQUAL=1.00]*[bias=3.00]*[target=2.00] | -0.1961 | 0.3564 | 1 | 1 | -1.397 | 1.0044 |
|  | [VIDEOQUAL=2.00]*[bias=1.00]*[target=1.00] | -0.8056 | 0.517 | 1 | 1 | -2.547 | 0.9357 |
|  | [VIDEOQUAL=2.00]*[bias=1.00]*[target=2.00] | 0.71 | 0.5025 | 1 | 1 | -0.982 | 2.4022 |
|  | [VIDEOQUAL=2.00]*[bias=2.00]*[target=1.00] | -0.1306 | 0.5153 | 1 | 1 | -1.866 | 1.6048 |
|  | [VIDEOQUAL=2.00]*[bias=2.00]*[target=2.00] | -0.8485 | 0.5007 | 1 | 1 | -2.535 | 0.8377 |
|  | [VIDEOQUAL=2.00]*[bias=3.00]*[target=1.00] | 0.07 | 0.4855 | 1 | 1 | -1.565 | 1.705 |
|  | [VIDEOQUAL=2.00]*[bias=3.00]*[target=2.00] | -0.6167 | 0.4914 | 1 | 1 | -2.272 | 1.0383 |
| [VIDEOQUAL=1.00]*[bias=2.00]*[target=1.00] | [VIDEOQUAL=1.00]*[bias=1.00]*[target=1.00] | -1.0266 | 0.334 | 1 | 0.139 | -2.151 | 0.0981 |
|  | [VIDEOQUAL=1.00]*[bias=1.00]*[target=2.00] | 0.4471 | 0.4227 | 1 | 1 | -0.977 | 1.8708 |
|  | [VIDEOQUAL=1.00]*[bias=2.00]*[target=2.00] | -0.272 | 0.4623 | 1 | 1 | -1.829 | 1.2851 |
|  | [VIDEOQUAL=1.00]*[bias=3.00]*[target=1.00] | -0.7377 | 0.3482 | 1 | 1 | -1.91 | 0.435 |
|  | [VIDEOQUAL=1.00]*[bias=3.00]*[target=2.00] | 0.251 | 0.4813 | 1 | 1 | -1.37 | 1.872 |
|  | [VIDEOQUAL=2.00]*[bias=1.00]*[target=1.00] | -0.3584 | 0.5402 | 1 | 1 | -2.178 | 1.4607 |
|  | [VIDEOQUAL=2.00]*[bias=1.00]*[target=2.00] | 1.1571 | 0.5262 | 1 | 1 | -0.615 | 2.9292 |
|  | [VIDEOQUAL=2.00]*[bias=2.00]*[target=1.00] | 0.3166 | 0.5385 | 1 | 1 | -1.497 | 2.13 |
|  | [VIDEOQUAL=2.00]*[bias=2.00]*[target=2.00] | -0.4014 | 0.5245 | 1 | 1 | -2.168 | 1.3652 |
|  | [VIDEOQUAL=2.00]*[bias=3.00]*[target=1.00] | 0.5171 | 0.51 | 1 | 1 | -1.201 | 2.2347 |
|  | [VIDEOQUAL=2.00]*[bias=3.00]*[target=2.00] | -0.1696 | 0.5157 | 1 | 1 | -1.906 | 1.5671 |
| [VIDEOQUAL=1.00]*[bias=2.00]*[target=2.00] | [VIDEOQUAL=1.00]*[bias=1.00]*[target=1.00] | -0.7547 | 0.4275 | 1 | 1 | -2.194 | 0.685 |
|  | [VIDEOQUAL=1.00]*[bias=1.00]*[target=2.00] | 0.7191 | 0.3325 | 1 | 1 | -0.401 | 1.8387 |
|  | [VIDEOQUAL=1.00]*[bias=2.00]*[target=1.00] | 0.272 | 0.4623 | 1 | 1 | -1.285 | 1.829 |
|  | [VIDEOQUAL=1.00]*[bias=3.00]*[target=1.00] | -0.4657 | 0.4183 | 1 | 1 | -1.874 | 0.943 |
|  | [VIDEOQUAL=1.00]*[bias=3.00]*[target=2.00] | 0.523 | 0.2898 | 1 | 1 | -0.453 | 1.4991 |
|  | [VIDEOQUAL=2.00]*[bias=1.00]*[target=1.00] | -0.0865 | 0.5196 | 1 | 1 | -1.836 | 1.6634 |
|  | [VIDEOQUAL=2.00]*[bias=1.00]*[target=2.00] | 1.4291 | 0.5051 | 1 | 0.308 | -0.272 | 3.1302 |
|  | [VIDEOQUAL=2.00]*[bias=2.00]*[target=1.00] | 0.5885 | 0.5179 | 1 | 1 | -1.156 | 2.3326 |
|  | [VIDEOQUAL=2.00]*[bias=2.00]*[target=2.00] | -0.1295 | 0.5034 | 1 | 1 | -1.825 | 1.5657 |
|  | [VIDEOQUAL=2.00]*[bias=3.00]*[target=1.00] | 0.789 | 0.4882 | 1 | 1 | -0.855 | 2.4333 |
|  | [VIDEOQUAL=2.00]*[bias=3.00]*[target=2.00] | 0.1023 | 0.4941 | 1 | 1 | -1.562 | 1.7664 |
| [VIDEOQUAL=1.00]*[bias=3.00]*[target=1.00] | [VIDEOQUAL=1.00]*[bias=1.00]*[target=1.00] | -0.2889 | 0.3479 | 1 | 1 | -1.461 | 0.8827 |
|  | [VIDEOQUAL=1.00]*[bias=1.00]*[target=2.00] | 1.1848 | 0.4224 | 1 | 0.332 | -0.238 | 2.6075 |
|  | [VIDEOQUAL=1.00]*[bias=2.00]*[target=1.00] | 0.7377 | 0.3482 | 1 | 1 | -0.435 | 1.9104 |
|  | [VIDEOQUAL=1.00]*[bias=2.00]*[target=2.00] | 0.4657 | 0.4183 | 1 | 1 | -0.943 | 1.8744 |
|  | [VIDEOQUAL=1.00]*[bias=3.00]*[target=2.00] | 0.9887 | 0.418 | 1 | 1 | -0.419 | 2.3966 |
|  | [VIDEOQUAL=2.00]*[bias=1.00]*[target=1.00] | 0.3793 | 0.521 | 1 | 1 | -1.375 | 2.1339 |
|  | [VIDEOQUAL=2.00]*[bias=1.00]*[target=2.00] | 1.8948^a^ | 0.5066 | 1 | 0.012 | 0.1888 | 3.6008 |
|  | [VIDEOQUAL=2.00]*[bias=2.00]*[target=1.00] | 1.0543 | 0.5193 | 1 | 1 | -0.695 | 2.8031 |
|  | [VIDEOQUAL=2.00]*[bias=2.00]*[target=2.00] | 0.3363 | 0.5048 | 1 | 1 | -1.364 | 2.0364 |
|  | [VIDEOQUAL=2.00]*[bias=3.00]*[target=1.00] | 1.2548 | 0.4897 | 1 | 0.687 | -0.395 | 2.9041 |
|  | [VIDEOQUAL=2.00]*[bias=3.00]*[target=2.00] | 0.5681 | 0.4956 | 1 | 1 | -1.101 | 2.2372 |
| [VIDEOQUAL=1.00]*[bias=3.00]*[target=2.00] | [VIDEOQUAL=1.00]*[bias=1.00]*[target=1.00] | -1.2777 | 0.4382 | 1 | 0.234 | -2.754 | 0.1982 |
|  | [VIDEOQUAL=1.00]*[bias=1.00]*[target=2.00] | 0.1961 | 0.3564 | 1 | 1 | -1.004 | 1.3965 |
|  | [VIDEOQUAL=1.00]*[bias=2.00]*[target=1.00] | -0.251 | 0.4813 | 1 | 1 | -1.872 | 1.3699 |
|  | [VIDEOQUAL=1.00]*[bias=2.00]*[target=2.00] | -0.523 | 0.2898 | 1 | 1 | -1.499 | 0.4531 |
|  | [VIDEOQUAL=1.00]*[bias=3.00]*[target=1.00] | -0.9887 | 0.418 | 1 | 1 | -2.397 | 0.4191 |
|  | [VIDEOQUAL=2.00]*[bias=1.00]*[target=1.00] | -0.6095 | 0.4925 | 1 | 1 | -2.268 | 1.0492 |
|  | [VIDEOQUAL=2.00]*[bias=1.00]*[target=2.00] | 0.9061 | 0.4772 | 1 | 1 | -0.701 | 2.5131 |
|  | [VIDEOQUAL=2.00]*[bias=2.00]*[target=1.00] | 0.0655 | 0.4907 | 1 | 1 | -1.587 | 1.718 |
|  | [VIDEOQUAL=2.00]*[bias=2.00]*[target=2.00] | -0.6525 | 0.4753 | 1 | 1 | -2.253 | 0.9484 |
|  | [VIDEOQUAL=2.00]*[bias=3.00]*[target=1.00] | 0.266 | 0.4593 | 1 | 1 | -1.281 | 1.8128 |
|  | [VIDEOQUAL=2.00]*[bias=3.00]*[target=2.00] | -0.4207 | 0.4656 | 1 | 1 | -1.989 | 1.1472 |
| [VIDEOQUAL=2.00]*[bias=1.00]*[target=1.00] | [VIDEOQUAL=1.00]*[bias=1.00]*[target=1.00] | -0.6682 | 0.5201 | 1 | 1 | -2.42 | 1.0833 |
|  | [VIDEOQUAL=1.00]*[bias=1.00]*[target=2.00] | 0.8056 | 0.517 | 1 | 1 | -0.936 | 2.5468 |
|  | [VIDEOQUAL=1.00]*[bias=2.00]*[target=1.00] | 0.3584 | 0.5402 | 1 | 1 | -1.461 | 2.1776 |
|  | [VIDEOQUAL=1.00]*[bias=2.00]*[target=2.00] | 0.0865 | 0.5196 | 1 | 1 | -1.663 | 1.8364 |
|  | [VIDEOQUAL=1.00]*[bias=3.00]*[target=1.00] | -0.3793 | 0.521 | 1 | 1 | -2.134 | 1.3754 |
|  | [VIDEOQUAL=1.00]*[bias=3.00]*[target=2.00] | 0.6095 | 0.4925 | 1 | 1 | -1.049 | 2.2682 |
|  | [VIDEOQUAL=2.00]*[bias=1.00]*[target=2.00] | 1.5155 | 0.4835 | 1 | 0.114 | -0.113 | 3.1439 |
|  | [VIDEOQUAL=2.00]*[bias=2.00]*[target=1.00] | 0.675 | 0.3987 | 1 | 1 | -0.668 | 2.0176 |
|  | [VIDEOQUAL=2.00]*[bias=2.00]*[target=2.00] | -0.043 | 0.431 | 1 | 1 | -1.495 | 1.4085 |
|  | [VIDEOQUAL=2.00]*[bias=3.00]*[target=1.00] | 0.8755 | 0.3548 | 1 | 0.898 | -0.32 | 2.0706 |
|  | [VIDEOQUAL=2.00]*[bias=3.00]*[target=2.00] | 0.1888 | 0.4368 | 1 | 1 | -1.282 | 1.6598 |
| [VIDEOQUAL=2.00]*[bias=1.00]*[target=2.00] | [VIDEOQUAL=1.00]*[bias=1.00]*[target=1.00] | -2.1837^a^ | 0.5056 | 1 | 0.001 | -3.887 | -0.481 |
|  | [VIDEOQUAL=1.00]*[bias=1.00]*[target=2.00] | -0.71 | 0.5025 | 1 | 1 | -2.402 | 0.9822 |
|  | [VIDEOQUAL=1.00]*[bias=2.00]*[target=1.00] | -1.1571 | 0.5262 | 1 | 1 | -2.929 | 0.615 |
|  | [VIDEOQUAL=1.00]*[bias=2.00]*[target=2.00] | -1.4291 | 0.5051 | 1 | 0.308 | -3.13 | 0.2721 |
|  | [VIDEOQUAL=1.00]*[bias=3.00]*[target=1.00] | -1.8948^a^ | 0.5066 | 1 | 0.012 | -3.601 | -0.189 |
|  | [VIDEOQUAL=1.00]*[bias=3.00]*[target=2.00] | -0.9061 | 0.4772 | 1 | 1 | -2.513 | 0.701 |
|  | [VIDEOQUAL=2.00]*[bias=1.00]*[target=1.00] | -1.5155 | 0.4835 | 1 | 0.114 | -3.144 | 0.1128 |
|  | [VIDEOQUAL=2.00]*[bias=2.00]*[target=1.00] | -0.8405 | 0.432 | 1 | 1 | -2.296 | 0.6145 |
|  | [VIDEOQUAL=2.00]*[bias=2.00]*[target=2.00] | -1.5585^a^ | 0.3448 | 1 | 0 | -2.72 | -0.397 |
|  | [VIDEOQUAL=2.00]*[bias=3.00]*[target=1.00] | -0.64 | 0.4142 | 1 | 1 | -2.035 | 0.755 |
|  | [VIDEOQUAL=2.00]*[bias=3.00]*[target=2.00] | -1.3267^a^ | 0.3602 | 1 | 0.015 | -2.54 | -0.114 |
| [VIDEOQUAL=2.00]*[bias=2.00]*[target=1.00] | [VIDEOQUAL=1.00]*[bias=1.00]*[target=1.00] | -1.3432 | 0.5184 | 1 | 0.631 | -3.089 | 0.4026 |
|  | [VIDEOQUAL=1.00]*[bias=1.00]*[target=2.00] | 0.1306 | 0.5153 | 1 | 1 | -1.605 | 1.8659 |
|  | [VIDEOQUAL=1.00]*[bias=2.00]*[target=1.00] | -0.3166 | 0.5385 | 1 | 1 | -2.13 | 1.4969 |
|  | [VIDEOQUAL=1.00]*[bias=2.00]*[target=2.00] | -0.5885 | 0.5179 | 1 | 1 | -2.333 | 1.1556 |
|  | [VIDEOQUAL=1.00]*[bias=3.00]*[target=1.00] | -1.0543 | 0.5193 | 1 | 1 | -2.803 | 0.6946 |
|  | [VIDEOQUAL=1.00]*[bias=3.00]*[target=2.00] | -0.0655 | 0.4907 | 1 | 1 | -1.718 | 1.587 |
|  | [VIDEOQUAL=2.00]*[bias=1.00]*[target=1.00] | -0.675 | 0.3987 | 1 | 1 | -2.018 | 0.6676 |
|  | [VIDEOQUAL=2.00]*[bias=1.00]*[target=2.00] | 0.8405 | 0.432 | 1 | 1 | -0.615 | 2.2956 |
|  | [VIDEOQUAL=2.00]*[bias=2.00]*[target=2.00] | -0.718 | 0.4102 | 1 | 1 | -2.1 | 0.6636 |
|  | [VIDEOQUAL=2.00]*[bias=3.00]*[target=1.00] | 0.2005 | 0.3418 | 1 | 1 | -0.951 | 1.3517 |
|  | [VIDEOQUAL=2.00]*[bias=3.00]*[target=2.00] | -0.4862 | 0.4176 | 1 | 1 | -1.893 | 0.9203 |
| [VIDEOQUAL=2.00]*[bias=2.00]*[target=2.00] | [VIDEOQUAL=1.00]*[bias=1.00]*[target=1.00] | -0.6252 | 0.5039 | 1 | 1 | -2.322 | 1.0717 |
|  | [VIDEOQUAL=1.00]*[bias=1.00]*[target=2.00] | 0.8485 | 0.5007 | 1 | 1 | -0.838 | 2.5348 |
|  | [VIDEOQUAL=1.00]*[bias=2.00]*[target=1.00] | 0.4014 | 0.5245 | 1 | 1 | -1.365 | 2.168 |
|  | [VIDEOQUAL=1.00]*[bias=2.00]*[target=2.00] | 0.1295 | 0.5034 | 1 | 1 | -1.566 | 1.8247 |
|  | [VIDEOQUAL=1.00]*[bias=3.00]*[target=1.00] | -0.3363 | 0.5048 | 1 | 1 | -2.036 | 1.3638 |
|  | [VIDEOQUAL=1.00]*[bias=3.00]*[target=2.00] | 0.6525 | 0.4753 | 1 | 1 | -0.948 | 2.2533 |
|  | [VIDEOQUAL=2.00]*[bias=1.00]*[target=1.00] | 0.043 | 0.431 | 1 | 1 | -1.409 | 1.4945 |
|  | [VIDEOQUAL=2.00]*[bias=1.00]*[target=2.00] | 1.5585^a^ | 0.3448 | 1 | 0 | 0.3972 | 2.7199 |
|  | [VIDEOQUAL=2.00]*[bias=2.00]*[target=1.00] | 0.718 | 0.4102 | 1 | 1 | -0.664 | 2.0996 |
|  | [VIDEOQUAL=2.00]*[bias=3.00]*[target=1.00] | 0.9185 | 0.391 | 1 | 1 | -0.398 | 2.2354 |
|  | [VIDEOQUAL=2.00]*[bias=3.00]*[target=2.00] | 0.2318 | 0.3442 | 1 | 1 | -0.927 | 1.3908 |
| [VIDEOQUAL=2.00]*[bias=3.00]*[target=1.00] | [VIDEOQUAL=1.00]*[bias=1.00]*[target=1.00] | -1.5437 | 0.4888 | 1 | 0.105 | -3.19 | 0.1023 |
|  | [VIDEOQUAL=1.00]*[bias=1.00]*[target=2.00] | -0.07 | 0.4855 | 1 | 1 | -1.705 | 1.5651 |
|  | [VIDEOQUAL=1.00]*[bias=2.00]*[target=1.00] | -0.5171 | 0.51 | 1 | 1 | -2.235 | 1.2006 |
|  | [VIDEOQUAL=1.00]*[bias=2.00]*[target=2.00] | -0.789 | 0.4882 | 1 | 1 | -2.433 | 0.8553 |
|  | [VIDEOQUAL=1.00]*[bias=3.00]*[target=1.00] | -1.2548 | 0.4897 | 1 | 0.687 | -2.904 | 0.3946 |
|  | [VIDEOQUAL=1.00]*[bias=3.00]*[target=2.00] | -0.266 | 0.4593 | 1 | 1 | -1.813 | 1.2808 |
|  | [VIDEOQUAL=2.00]*[bias=1.00]*[target=1.00] | -0.8755 | 0.3548 | 1 | 0.898 | -2.071 | 0.3195 |
|  | [VIDEOQUAL=2.00]*[bias=1.00]*[target=2.00] | 0.64 | 0.4142 | 1 | 1 | -0.755 | 2.035 |
|  | [VIDEOQUAL=2.00]*[bias=2.00]*[target=1.00] | -0.2005 | 0.3418 | 1 | 1 | -1.352 | 0.9506 |
|  | [VIDEOQUAL=2.00]*[bias=2.00]*[target=2.00] | -0.9185 | 0.391 | 1 | 1 | -2.235 | 0.3984 |
|  | [VIDEOQUAL=2.00]*[bias=3.00]*[target=2.00] | -0.6867 | 0.3433 | 1 | 1 | -1.843 | 0.4693 |
| [VIDEOQUAL=2.00]*[bias=3.00]*[target=2.00] | [VIDEOQUAL=1.00]*[bias=1.00]*[target=1.00] | -0.857 | 0.4946 | 1 | 1 | -2.523 | 0.8088 |
|  | [VIDEOQUAL=1.00]*[bias=1.00]*[target=2.00] | 0.6167 | 0.4914 | 1 | 1 | -1.038 | 2.2718 |
|  | [VIDEOQUAL=1.00]*[bias=2.00]*[target=1.00] | 0.1696 | 0.5157 | 1 | 1 | -1.567 | 1.9064 |
|  | [VIDEOQUAL=1.00]*[bias=2.00]*[target=2.00] | -0.1023 | 0.4941 | 1 | 1 | -1.766 | 1.5618 |
|  | [VIDEOQUAL=1.00]*[bias=3.00]*[target=1.00] | -0.5681 | 0.4956 | 1 | 1 | -2.237 | 1.101 |
|  | [VIDEOQUAL=1.00]*[bias=3.00]*[target=2.00] | 0.4207 | 0.4656 | 1 | 1 | -1.147 | 1.9886 |
|  | [VIDEOQUAL=2.00]*[bias=1.00]*[target=1.00] | -0.1888 | 0.4368 | 1 | 1 | -1.66 | 1.2822 |
|  | [VIDEOQUAL=2.00]*[bias=1.00]*[target=2.00] | 1.3267^a^ | 0.3602 | 1 | 0.015 | 0.1137 | 2.5397 |
|  | [VIDEOQUAL=2.00]*[bias=2.00]*[target=1.00] | 0.4862 | 0.4176 | 1 | 1 | -0.92 | 1.8927 |
|  | [VIDEOQUAL=2.00]*[bias=2.00]*[target=2.00] | -0.2318 | 0.3442 | 1 | 1 | -1.391 | 0.9272 |
|  | [VIDEOQUAL=2.00]*[bias=3.00]*[target=1.00] | 0.6867 | 0.3433 | 1 | 1 | -0.469 | 1.8427 |
| Pairwise comparisons of estimated marginal means based on the original scale of dependent variable confidence | | | | | | | |
|  | | | | | | | |
